# Data sharing and publishing in the field of neuroimaging

**DOI:** 10.1186/2047-217X-1-9

**Published:** 2012-07-12

**Authors:** Janis L Breeze, Jean-Baptiste Poline, David N Kennedy

**Affiliations:** 1 International Neuroinformatics Coordinating Facility, Stockholm, Sweden; 2 Neurospin, CEA, Gif-sur-Yvette, France; 3 University of Massachusetts Medical Center, Worcester, MA, USA

**Keywords:** Neuroimaging, Neuroscience, Data sharing, Data publishing, Standards

## Abstract

There is growing recognition of the importance of data sharing in the neurosciences, and in particular in the field of neuroimaging research, in order to best make use of the volumes of human subject data that have been acquired to date. However, a number of barriers, both practical and cultural, continue to impede the widespread practice of data sharing; these include: lack of standard infrastructure and tools for data sharing, uncertainty about how to organize and prepare the data for sharing, and researchers’ fears about unattributed data use or missed opportunities for publication. A further challenge is how the scientific community should best describe and/or reference shared data that is used in secondary analyses. Finally, issues of human research subject protections and the ethical use of such data are an ongoing source of concern for neuroimaging researchers.

One crucial issue is how producers of shared data can and should be acknowledged and how this important component of science will benefit individuals in their academic careers. While we encourage the field to make use of these opportunities for data publishing, it is critical that standards for metadata, provenance, and other descriptors are used. This commentary outlines the efforts of the International Neuroinformatics Coordinating Facility Task Force on Neuroimaging Datasharing to coordinate and establish such standards, as well as potential ways forward to relieve the issues that researchers who produce these massive, reusable community resources face when making the data rapidly and freely available to the public. Both the technical and human aspects of data sharing must be addressed if we are to go forward.

## Background

With the worldwide push for more open science and data sharing [1], it is an ideal time to consider the current state of data sharing in neuroscience, and in particular neuroimaging research. A huge amount of neuroimaging data has been acquired around the world; a recent literature search on PubMed led to an estimate of 12 000 datasets or 144 000 scans (around 55 petabytes of data) over the past 10 years, but only a few percent of such data is available in public repositories. Over the past two years, the International Neuroinformatics Coordinating Facility (http://www.incf.org) has investigated barriers to data sharing through task force working groups and public workshops, and has identified a number of roadblocks, many of which are readily addressable, that impede researchers from both sharing and making use of existing shared data. These include a lack of simple tools for finding, uploading, and downloading shared data; uncertainty about how to best organize and prepare data for sharing, and concerns about data attribution. Many researchers are also wary of data sharing because of confusion institutional human research subject protection and the ethical use of such data [2].

Several journals have played a key role in the trend toward having data available for reviewers or readers of a peer-reviewed paper. The *Journal of Cognitive Neuroscience* was a pioneer in this context, and while the project was probably too ambitious for the capacity of the tools and for the size of the team, the trend for data “on demand” has remained with several high ranked journals. The requirement to share data, and the infrastructure to support this data sharing present numerous associated technical difficulties and costs to the journal. Nonetheless, in the future it may be that both data and computational tools will be made available in some new form of ‘supplementary material’ or associated data warehouse to help track the shared data and it’s provenance.

A growing and crucial issue is how producers of shared data can and should be acknowledged by third parties who publish papers based on this data. Without such acknowledgement, very little data will ever be shared. A number of journals have launched a new type of articles devoted to the description and/or publication of original datasets [3,4]. The benefit of using a publication to ‘mark’ a data release is that credit and reuse are fairly easily tracked with traditional citation and impact metrics. With its ability to host large datasets, *GigaScience* offers neuroimaging researchers another option to store and share their data, and provides such datasets a digital object identifier.

## Discussion

Given the challenges in carrying out controlled research with human subjects—it simply isn’t possible or ethical to treat people like monkeys—questions in human biomedical research are generally best studied with very large datasets. While meta-analyses offer a possible workaround by aggregating the published results of studies, this is obviously less desirable than working with the raw data themselves. To give one example, though functional neuroimaging research typically reports activation locations using coordinates, Salimi-Khorshidi and colleagues [5] recently showed that the consistency between a study using the original contrast maps and those derived from the coordinates alone was poor.

There is little doubt that more and more neuroimaging data will be shared. For example, increased attention to the importance of reproducible research [6] has helped to encourage that data and analysis tools are made available as supplementary material at the time of publication. Another impetus is the need for many projects to gather data and communicate with collaborators. Whenever the scientific questions require a large number of scans, longitudinal data, or a very specific patient population that cannot be recruited at one site, researchers from consortia need standard tools to share and curate data and computational tools.

The INCF Neuroimaging Datasharing Task Force found that even where enthusiasm for data sharing exists, it is tempered by a number of technical issues that prevent the average neuroimaging researcher from participating fully in the data sharing community. In particular, a lack of standards, recommendations, and interoperable and easy-to-use tools for sharing is lacking. In an attempt to improve this situation, the group is working on four projects to be completed by the end of 2012. In brief, (1) a “One-Click Share Tool” will allow researchers to upload MRI data (in DICOM or NIFTI format) to a database hosted at INCF. A quality control check will provide the uploader with feedback about their data; (2) Building on previous efforts, a neuroimaging data description schema and common application programming interface (API) will facilitate communication among databases with different data models; (3) A mechanism to capture related data under a single container will be introduced; (4) Metadata and the results of processing streams will be automatically stored to a database, including the previously described quality control workflows and any processed data and metadata.

While the lack of tools is an obvious barrier, it is one that we feel can be readily addressed by efforts such as that of INCF and similar initiatives [7-9]. A greater challenge will be the current academic and funding framework in which most researchers exist, which equates career advancement with some count of peer-reviewed publications. Given this climate, it is a great step forward for the community that peer-reviewed journals are now offering an article type devoted to the description and publication of data, along with recommendation of organizations such as DataCite. This follows similar journal initiatives to publish papers on software code and technology methods, and signifies a stronger valuation of the computational and technical work that makes up a large part of modern biomedical research.

Data papers should describe in detail how the data was acquired, with which goals and constraints, an assessment of their quality, how they have and how they can or should be reused, how to get access, give feedback and credit. Datasets are technical and critical building blocks of science and should be recognized as such by high impact and heavy citation, ensuring that creators of data are appropriately acknowledged for their work.

## Conclusions

The impact of widespread data sharing on our field should be enormous—it will provide better training opportunities to students by enabling them to work with large amounts of real data; it will alter our interpretation and understanding of the variability of brain function; it will lead to better reporducibility and stronger data analysis and interpretations; and it will lead to new methods and tools for analyzing massive datasets.

While we encourage the field to make use of opportunities for data publishing, we realize that standards for metadata, provenance, and other descriptors are critical. INCF’s Task Force on Data Sharing looks forward to working with the community to converge on such standards. All tools and databases provided hosted by INCF are open-access and without a doubt strengthened by community feedback.

## Competing interests

Jean-Baptiste Poline sits on the INCF Governing Board.

## Authors’ contributions

All authors contributed equally to the conception and writing of this manuscript. All authors read and approved the final manuscript.

## References

[B1] Uhlir PF Schröder P Open Data for Global Science Data Sci J 2007 6 OD36 OD53

[B2] Kehagia AA Tairyan K Federico C Glover GH Illes J More Education, Less Administration: Reflections of Neuroimagers’ Attitudes to Ethics Through the Qualitative Looking Glass Sci Eng Ethics 2011 May 28 [Epub ahead of print]. 10.1007/s11948-011-9282-2 21626219

[B3] Kennedy DN Ascoli GA De Schutter E Next Steps in Data Publishing Neuroinformatics 2011 9 317 320 10.1007/s12021-011-9131-0

[B4] Hrynaszkiewicz I A call for BMC Research Notes contributions promoting best practice in data standardization, sharing and publication BMC Res Notes 2010 3 235 10.1186/1756-0500-3-235 20813027 PMC2940773

[B5] Salimi-Khorshidi G Smith SM Keltner JR Wager TD Nichols TE Meta-analysis of neuroimaging data: a comparison of image-based and coordinate-based pooling of studies Neuroimage 2009 45 810 823 10.1016/j.neuroimage.2008.12.039 19166944

[B6] Donoho DL An invitation to reproducible computational research Biostatistics 2010 11 385 388 10.1093/biostatistics/kxq028 20538873

[B7] Keator DB Wei D Gadde S Derived Data Storage and Exchange Workflow for Large-Scale Neuroimaging Analyses on the BIRN Grid Front Neuroinformatics 2009 3 30 10.3389/neuro.11.030.2009 PMC2759340 19826494

[B8] Marcus DS Harwell J Olsen T Informatics and data mining tools and strategies for the human connectome project Front Neuroinformatics 2011 5 4 10.3389/fninf.2011.00004 PMC3127103 21743807

[B9] Yarkoni T Poldrack RA Nichols TE Van Essen DC Wager TD Large-scale automated synthesis of human functional neuroimaging data Nat Methods 2011 8 665 670 10.1038/nmeth.1635 21706013 PMC3146590

